# Factor contribution to fire occurrence, size, and burn probability in a subtropical coniferous forest in East China

**DOI:** 10.1371/journal.pone.0172110

**Published:** 2017-02-16

**Authors:** Tao Ye, Yao Wang, Zhixing Guo, Yijia Li

**Affiliations:** 1 Academy of Disaster Reduction and Emergency Management, Ministry of Civil Affairs and Ministry of Education, Beijing Normal University, Beijing, China; 2 Faculty of Geographical Science, Beijing Normal University, Beijing, China; 3 Catastrophe Research Center, Beijing Normal University, Beijing, China; 4 National Marine Hazard Mitigation Service, State Oceanic Administration People’s Republic of China, Beijing, China; Universidade de Vigo, SPAIN

## Abstract

The contribution of factors including fuel type, fire-weather conditions, topography and human activity to fire regime attributes (e.g. fire occurrence, size distribution and severity) has been intensively discussed. The relative importance of those factors in explaining the burn probability (BP), which is critical in terms of fire risk management, has been insufficiently addressed. Focusing on a subtropical coniferous forest with strong human disturbance in East China, our main objective was to evaluate and compare the relative importance of fuel composition, topography, and human activity for fire occurrence, size and BP. Local BP distribution was derived with stochastic fire simulation approach using detailed historical fire data (1990–2010) and forest-resource survey results, based on which our factor contribution analysis was carried out. Our results indicated that fuel composition had the greatest relative importance in explaining fire occurrence and size, but human activity explained most of the variance in BP. This implies that the influence of human activity is amplified through the process of overlapping repeated ignition and spreading events. This result emphasizes the status of strong human disturbance in local fire processes. It further confirms the need for a holistic perspective on factor contribution to fire likelihood, rather than focusing on individual fire regime attributes, for the purpose of fire risk management.

## Introduction

Forest fire represents a significant threat to ecological and social systems [1], causing escalating social, eco-environmental and fiscal costs along with the changing climate [2,3]. It is a result of complex human–environment interactions involving fuel, climate and fire-weather conditions, and topography [4,5]. Climate and weather conditions influence the fire regime by determining the fuel distribution and the occurrence of fire [6–8]. Topography alters the microclimate and local fuel distributions [9]. Human activities also intervene in local fire regimes by changing ignition patterns [6], altering the fuel distribution, and suppressing fires [10,11]. The influence of these factors on individual fire regime attributes, e.g., fire occurrence, size distribution and severity, has been intensively discussed in the literature. For fire occurrence, efforts have been made to understand its key drivers and improve its prediction [6,12–16]. In fire size analysis, researchers focused on the contribution of type and continuity of vegetation [17], fire weather and topographic factors that controls the rate of fire spread and duration of favorable conditions, and human activity in fire suppression and extinguishment [18]. The effect of spatial scale on the dominant controlling factors are also intensively discussed [19]. Factor contribution to the occurrence, size distribution and severity of forest fires may differ substantially even for the same region and consequently comparative studies are carried out to improve understanding of regional fire regimes [7].

Understanding regional regimes is key for present-day fire prevention and management [20]. Recently, the burn probability (BP) of forest fire has been provided to fire management agencies [21] as a move from hazard management to risk management [22]. This provides a measure of the likelihood of fire based on numerical simulation of fire ignition and spreading [23–26], rather than focusing on individual events. It also provides critical information for fire managers to make holistic risk-management decisions. Nevertheless, the focus on the separate effects of fuel, fire-weather conditions, topography and human influence on individual fire regimes is insufficient to reveal the major drivers of fire risk [8]. On the one hand, some factors may contribute positively to one regime component but negatively to another. For instance, human activities may substantially increase the ignition density, but also provide a greater capacity for fire suppression and the control of fire size and severity [19]. On the other hand, the distribution of the BP is a non-linear function of the various factors affecting fire regimes [27,28]. The non-linear nature of the system could amplify certain factors’ contributions while attenuating others.

Our central purpose was to evaluate and compare the relative contribution of fuel composition, topography, and human activity to fire occurrence, size, and BP in a subtropical coniferous forest region in East China. The study area is a county on the border of Zhejiang and Fujian Provinces [19,29–31]. Zhejiang and Fujian are among the top three provinces with the highest forest coverage rate in China, the top eight provinces with the largest plantation, and also among the provinces with the highest forest productions output [32]. During the period 1998–2013, the annual average forest area burned in this region was the third largest nationwide, following Heilongjiang and Inner Mongolia [33]. Most of the forest fires were ignited due to human activity. The rapid cycle of forestry production mixed with the protection of natural forest, together with strong human disturbance, makes for a typical forest fire regime representative of East and South China. We started our analysis by modeling and predicting the fire ignition probability and size distribution. The fire likelihood as measured by the BP was then derived through simulation of a large number of individual fires based on the estimated ignition and size parameters. A detailed data set containing forest-resource survey results and a historical fire data supported this analysis. Finally, the relationship between the BP results and the potential influencing factors, and the relative contribution of those factors, were discussed.

## Materials

### Study area

**Fig 1:**
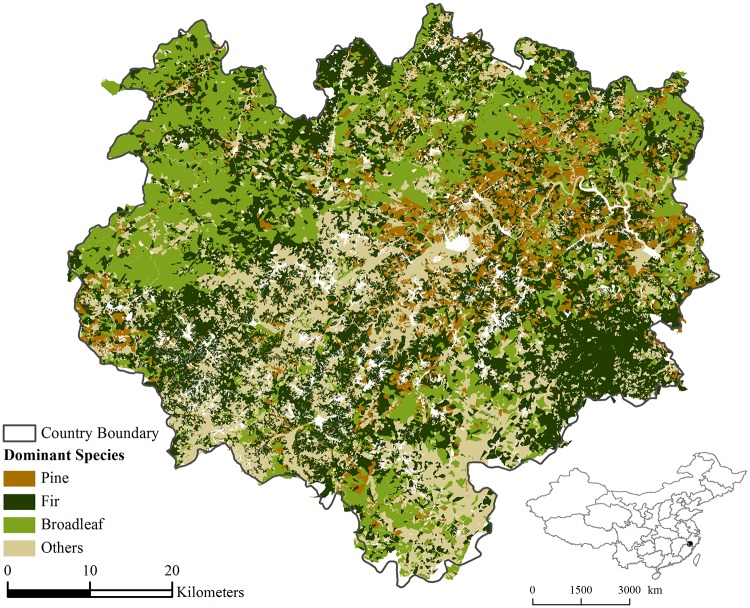
The study area and the distribution of fuel type in terms of dominant species.

The study area is Longquan County in Zhejiang Province, China (118.70°E–119.42°E and 27.70°N – 28.33°N, Fig 1). It has a total land area of 3,059 km^2^, and the elevation ranges from 168 to 1922 m. This area has a subtropical monsoon climate with hot and rainy summers, and mild and dry winters. The annual average precipitation is about 1700 mm, 80% of which occurs between March and September. The mean annual temperature is about 17.6°C, while the maximum temperature can be higher than 40°C in the summer.

The climax vegetation is subtropical coniferous forest (46% of the total forest area in this region), mixed with subtropical deciduous broadleaf forest, subtropical evergreen broadleaf forest (29% of the total forest area) and subtropical and tropical bamboo (16% of the total forest area). The dominant tree species in the subtropical coniferous forest are *Pinus massoniana*, *Pinus taiwanensis*, *Pinus elliottii*, *Cupressus funebris*, *Taxus chinensis* and *Tsuga chinensis*. The dominant tree species in the broadleaf forests are *Quercus acutissima*, *Phoebe zhennan* and *Eucalyptus robusta*. *Paulownia kawakamii* and other softwood species, such as *Populus L*., *Paulownia Sieb*. *et Zucc*., etc, also exist. Broadleaf forests are planted as fire-resistant species in local forest planning. Bamboo and other economic forests occupy 25% of the total forest area, and are also expected to be relative low flammability.

### Data

Data used in this study included historical fire data, fuel distribution, fire weather, topography and human activity (Table 1).

**Table 1:** Dependent and exploratory variables included in this study.

Variables	Description	Mean±Sd
**Historical fire data**		
Fire (Fire size: ha)	Including information about ignitions coordinates, exact date of occurrence, time of suppression, fire size, and the cause of fire.	(Fire size) 5.41±10.31
**Fuel distribution**		
Proportion of pine (%)	Percentage of pine in a forest stand.	14.13±25.09
Proportion of fir (%)	Percentage of fir in a forest stand.	38.64±37.33
Proportion of broadleaf (%)	Percentage of broadleaf in a forest stand.	18.00±29.56
Proportion of others (%)	Percentage of others in a forest stand.	29.23±41.32
Dominant species age (yr)	The average age of dominant species in a forest stand.	17.92±13.72
**Fire Weather**		
Daily maximum temperature (°C)	Daily maximum temperature on the ignition/non-ignition day.	24.87±7.97
Daily average temperature (°C)	Daily mean temperature on the ignition/non-ignition day.	16.92±7.26
Daily average relative humidity (%)	Daily mean relative humidity on the ignition/non-ignition day.	73.16±10.61
Daily precipitation (mm)	Daily cumulative precipitation on the ignition/non-ignition day.	2.90±9.80
Wind direction	Wind direction of the maximum wind velocity on the ignition/non-ignition day, which is divided into 16 directions: 1-N; 2-NNE; 3-NE; 4-ENE; 5-E; 6-ESE; 7-SE; 8-SSE; 9-S; 10-SSW; 11-SW; 12-WSW; 13-W; 14-WNW; 15-NW; 16-NNW.	7.30±4.17
Maximum wind velocity (m/s)	The maximum wind velocity on the ignition/non-ignition day.	1.15±0.83
**Topography**		
Elevation (m)	Mean elevation in a forest stand.	640.66**±**325.9
Slope (degrees)	Mean slope in a forest stand.	18.29**±**9.71
Aspect (degrees)	Mean aspect in a forest stand.	182.61**±**98.47
**Human activity**		
Population density (people/km^2^)	The number of people in 1 km^2^.	226**±**1375
Distance to nearest road (km)	Distance from ignition/non-ignition to the nearest road.	5.03**±**5.56
Distance to nearest settlement (km)	Distance from ignition/non-ignition to the nearest settlement.	9.05**±**7.03

#### Historical fire data

**Fig 2:**
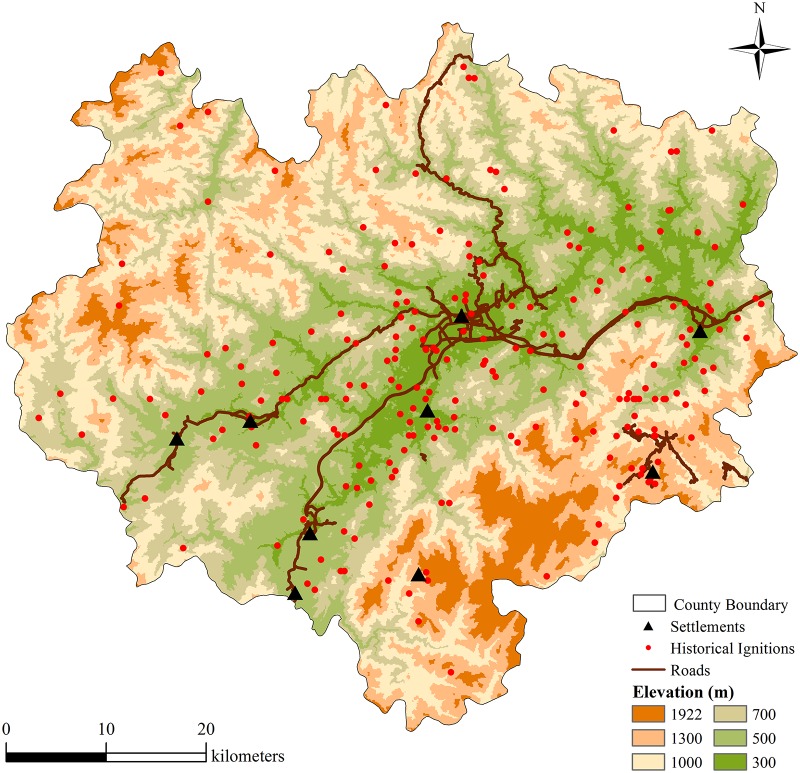
Elevation, historical ignitions (1991–2010), settlements and roads in Longquan.

We obtained historical fire data from Zhejiang Provincial Forest Fire Prevention Agency. The data set contains a total of 258 fires in Longquan during the period 1991–2010 (Fig 2), among which 225 were valid for analysis. This data is the only data available for the period 1991–2010 since the weather conditions of the study area strongly constrain the availability of satellite imagery. It contains precise records of ignitions coordinates, exact date of occurrence, time of suppression, fire size, and the cause of fire.

Descriptive statistics show that the total burned area during the 20-year period was 1217.43 ha, while the minimum and maximum fire sizes were 0.3 ha and 80.3 ha, respectively. Seasonally, winter and spring (December to May) were more fire-prone than other seasons (191 out of 225). Out of the 225 fire events, 182 (71.4%) were confirmed to be the result of human activity. Only one event was caused by lightning ignition (S1 Fig). Historical fire locations showed strong connections with roads, settlements and places with lower elevation (Fig 2). Therefore, the fire regime in this region is markedly different from those found in Northeast China, where fires are infrequent but intense with return period of more than hundreds of years [19], and lightning-caused fires takes a larger share (43%) of total fire occurrence than human-caused fires (21%) [6].

#### Fuel distribution

A forest stand map of the study area for the year 2008 was obtained from the local county forest administration (Forestry Administration of Longquan County, http://lyj.longquan.gov.cn, Fig 1). These data came from the seventh synthesis report of the national forest inventory survey, starting in 1973 and reporting every five years. In this survey system, forests were recorded and digitally mapped into numerous polygons (termed “subcompartments;” GB/T 26424–2010). Our study area contains 41,669 subcompartment polygons in total. The sizes of the subcompartments vary substantially, ranging from 0.01 to 305.07 ha, with a mean, median and standard deviation of 6.99 ha, 5.19 ha, and 8.85 ha, respectively.

More than 50 attributes were provided in the attribute tables to thoroughly describe the forest in each subcompartment. Attributes essential to discussing fire occurrence and size were mostly included, e.g., forest category, species composition (% area of major tree species), dominant species, dominant species age, etc. To conduct factor contribution analysis without too many explanatory variables, the 56 tree species reported in the study area were grouped into four main forest types: pine, fir, broadleaf and others. The criteria of grouping tree species is Flora of China (http://foc.eflora.cn/), which has a complete list of all plants in China and a sound classification system of them according to taxonomy. This reference was used as a look-up table to get the grouping results.

#### Fire weather

For fire weather, we used Climate Forecast System Reanalysis (CFSR) hourly data from the period 1991–2010 provided by the National Centers for Environmental Prediction (NECP, http://rda.ucar.edu/). These are gridded data, in which the temperature values are provided with a spatial resolution of 0.312° × 0.312°, and the other values with a resolution of 0.5° × 0.5°. This data set was chosen as the CFSR can provide continuous spatial data for the whole study area for the period 1991–2010 resolution. Otherwise, data can be obtained from only two national reference meteorological stations within the study area. Several important indices were extracted from the CFSR data set and converted into daily values: daily average and maximum temperature, daily average relative humidity, daily precipitation, wind direction and maximum wind velocity.

#### Topography

Topographic data, including the widely used variables of elevation, aspect, and slope, were derived from digital elevation model data (DEMs; 90 m × 90 m) provided by the NASA Shuttle Radar Topographic Mission (SRTM, http://srtm.csi.cgiar.org/). Slope was expressed in degrees, while aspect varied from 1 to 360.

#### Human activity

Three variables were considered in our analysis to represent local human activity intensity, as suggested by several previous studies, including population density, the distance to nearest road and the distance to nearest settlement [16,19]. Gridded population distribution maps (1 km × 1 km) were obtained from Oak Ridge National Laboratory (http://web.ornl.gov/sci/landscan/). The distances from each subcompartment to the nearest road and settlement were calculated based on spatial analysis using a road system map (Open Street Map; http://www.openstreetmap.org/) and settlements distribution map (National Geomatics Center of China, http://ngcc.sbsm.gov.cn/). Both road system map and settlements distribution map are vector data.

## Methods

### Fire occurrence analysis

We used binary logistic regression analysis to identify the significant factors influencing fire ignition/occurrence [14,21,34,35]. The logistic connection function allows a bounded estimation of ignition probability, which is not possible with linear regression. It contains a dichotomous response variable (1/0, ignited/not ignited) and various explanatory variables to accommodate the contributions of various factors, such as fuel, fire weather, topography and human activity. Explanatory variables were selected based on the suggestions from the existing literature, e.g., [14,16,21,28,30]. In total, 14 variables were considered, including fuel type attributes (proportion of pine, fir, broadleaf, and others in each subcompartment; dominant species age), local topography (elevation, slope and aspect), weather on the ignition day (daily precipitation, daily average temperature and daily average relative humidity) and human activity (population density, distance to nearest road, distance to nearest settlement).

To apply the binary logistic regression, we first randomly generated a number of non-ignition points similar to the number of ignition points, using the mean-nearest-neighbor-distance method [36]. The mean nearest-neighbor distance of the 225 ignitions in the study area was 1467.39 m. In total, 227 random non-ignitions were generated. Before we ran the logistic regression, Pearson correlation analysis was applied to the potential explanatory variables mentioned. Highly significantly correlated variables (Pearson Correlation Coefficient > 0.4) were restrained to enter the model simultaneously to avoid possible multicollinearity in the regression analysis.

The binary logistic regression model was fitted in R v3.2.4. To measure the model performance, Cox & Snell R^2^, Nagelkerke R^2^ and the area under the curve (AUC) of the receiver operating characteristic (ROC) plot were calculated to show the goodness-of-fit.

### Fire size analysis

Linear regression models were first applied to explain fire size with the same set of explanatory variables. Different combinations of explanatory variables were considered. Unfortunately, these models did not show good fits of the observed data, indicating a strongly non-linear relationship among the fire size and explanatory variables. Consequently, we used random forest (RF) to train and predict the fire sizes. RF is a machine-learning (ML) algorithm that derived from Classification and Regression Tree (CART) and usually outperform CART at both classification [37] and regression [38]. It also has shown promising capability in explaining regional fire regimes [13,39,40]. RF produces thousands of trees which allows each pixel to be assigned a more refined value. The accuracy of a random forest depends on the strength of the individual tree classifiers and a measure of the dependence between them [41]. Compared with the boosted decision tree approach, the predictive power of RF mostly lies in its bootstrap sampling and therefore typically requires deep trees [42]. It uses out-of-bag (OOB) samples randomly selected from the observations to calculate the error rates independently. Therefore, no test data nor cross-validation are needed. However, the output of RF does not provide regression coefficients or confidence intervals like other linear regression models, due to the complicated ensemble of many trees. Instead, variable importance measures, e.g., the mean decrease in accuracy (% IncMSE) and total variance explained, can be used to select variables and verify model performance [43].

The training with RF was carried out to determine two essential parameters: the number of variables to try at each split (*mtry*), and the total number of trees in the forest (*ntree*). The parameter *mtry* can be optimized by tuning the RF model, which starts at its default value ([total number of variables / 3] for regression analysis) and stops at the value that derives the minimum OOB error rate. The parameter *ntree* was set at 1000 for the initial value. Model fitting indicated that this value was sufficiently large to guarantee error convergence. In order to reduce stochasticity in the outcomes, we created an ensemble of 10 RF models. For each model, 150 observations (approximately 2/3 of the sample size) were randomly selected for training, and the rest were used for the OOB test. The final results of RF were the average of 10 models.

### Burn probability modeling and analysis

The fine-scale BP modeling involves the repetition of a very large number (> 10^4^) of single fire simulations. Three important steps are involved in simulating a single forest fire event: ignitions, spreading behavior, and suppression. The simulation of ignitions requires a highly consistent spatial-temporal pattern between the random ignitions generated and the historical fire data. Then it is required to obtain the actual date and location of ignitions. For the time of ignition, we analyzed the annual, seasonal, and monthly fire frequencies and generated random numbers of monthly ignitions (S2 Fig). Then, the date for each ignition within a month was randomly drawn from the days of the month. Spatially, the specific location of an ignition point was generated using Monte Carlo approach based on ignition probability maps, which can be derived with the ignition probability regression results and factor data inputs. The requested fire weather input for a given ignition day was randomly drawn from the historical weather of the neighboring 10 days of the past 20 years. With the ignition map, random ignitions can be obtained with the acceptance-rejection method [44]. The acceptance-rejection method is a type of Monte Carlo method based on the observation that to sample a random variable one can perform a uniformly random sampling of the 2D Cartesian graph, and keep the samples in the region under the graph of its density function [45].

For fire spreading behavior, there are plenty of models with parameters derived from historical fire cases [46], best known among them being the Rothermel model [47], CFFDRS model [48] and McArthur model [49]. The application of the models requires consistent local fuel system and fire regimes. To this end, we employed a surface fire spreading model as it was used [50], *R* = *R*_0_·*K*_*f*_·*K*_*w*_·*K*_*t*_, tailored for Chinese forest systems. *R* is the fire spreading speed (m/min). The initial speed *R*_0_ (m/min) is determined by the daily maximum temperature *T* (°C), wind speed *v* (m/s), and relative humidity *h*, *R*_0_ = *aT* + *bv* + *c*(1 − *h*) − *d*, with empirically estimated parameters *a* = 0.03, *b* = 0.05, *c* = 0.01 and *d* = 0.3. The fuel parameter *K*_*f*_ ranges from 0.8 to 1.8 by fuel type. These parameters were estimated from more than 100 fires in Chinese forests [50]. The effects of wind speed *v* and slope *ϕ* were calculated as *K*_*w*_ = exp(0.1783*v*) and *K*_*t*_ = exp(3.533(tan*ϕ*)^1.2^) [51].

The spatial spreading behavior was modeled using the cellular automation algorithm [52,53]. Cellular automata are models that assume space, state and time discrete. Each cell has a neighborhood, set of internal states variables and a local rule that describes the transition between the states variables and define the future state as a function of the cell present state and the neighborhood present states [54]. All input data, including fuel type, weather, topography and human activity, were transformed/resampled into 200 m resolution grids. Fuel composition, topography and human activity were considered static for each given grid in the simulation. The requested fire weather input for a given ignition day was again randomly drawn from the historical weather of the neighboring 10 days of the past 20 years. The simulation was replicated for 10,000 runs, each run contains 365 single fire days and in total 112,968 individual fires were simulated (S3 and S4 Figs). The simulation was coded with C++ language and compiled with Visual Studio 2016.

For the suppression of fire, the RF model for fire size trained above was used to decide the termination. Given the time and location of a simulated ignition, its fire size was derived from the RF prediction plus a random component derived from the RF fitting residuals describing the random error (S5 Fig). Two methods were applied to validate the historical fire size and simulated results. First we compared the historical and simulated empirical cumulative distribution of individual fire sizes by a two-sample Kolmogorov–Smirnov test. Then the annual average area burned in the historical data and the simulated result were compared.

Based on the BP results, the relative importance of the factors for the BP can be derived, and a linear regression approach was employed. In linear regression, the relative contribution of a single explanatory variable can be measured by the change in total variance explained by adding it to or removing it from the model. When regressors are uncorrelated, each regressor’s contribution is just the R^2^ from univariate regression. When variables are correlated, the change in the R^2^ metric is usually sensitive to the order in which the variables of interest are added/removed. In response, many decomposition methods have been proposed. The R package *relaimpo* implements six different methods for assessing the relative importance in linear regression. Among them, the *lmg* [55] metrics yielding averaged sequential changes in R^2^ was strongly suggested [56,57], particularly for the purpose of decomposing R^2^. We followed this suggestion and the analysis was carried out with the *relaimpo* package in R v3.2.4.

## Results

### Factor contribution to fire occurrence

In final binary logistic regression model, 9 variables were considered (Table 2). The proportion of other tree species in each subcompartment was retained as it holds perfect collinearity with proportions of pine, fir and broad leaf. Precipitation, elevation, local population density and distance to nearest road were not included in the model due to correlation coefficient over 0.4. The regression results yielded a Cox & Snell R^2^ and Nagelkerke R^2^ of 0.339 and 0.452, respectively. The area under the curve (AUC) of the receiver operating characteristic (ROC) plot is 84.95%, indicating a good model performance [14].

**Table 2:** Results of the binary logistic regression model.

Variables	B	S.E.	Wald	Sig.	Exp(B)
Constant	10.304	1.132	82.877	0.000	/
Proportion of pine (%)	0.881	0.509	2.999	0.083	2.414
Proportion of fir (%)	0.858	0.375	5.242	0.022	2.358
Proportion of broadleaf (%)	-0.615	0.526	1.366	0.243	0.541
Dominant species age (yr)	-0.034	0.012	7.890	0.005	0.967
Slope (degrees)	-0.027	0.014	3.766	0.052	0.974
Aspect (degrees)	-0.002	0.001	1.787	0.181	0.998
Daily average temperature (°C)	-0.075	0.017	19.461	0.000	0.928
Daily average relative humidity (%)	-0.101	0.013	56.206	0.000	0.904
Distance to nearest settlement (km)	-0.075	0.020	13.802	0.000	0.928

Where B, S.E., Wald, Sig. and Exp(B) represent regression coefficients, standard error of regression coefficients, Wald chi-square value, significance value and odds ratio, respectively. Additional estimation results using generalized adding models with incorporating interaction items are also provided in S1 Text.

Most of the variables show a statistically significant contribution to the ignition probability. In the fuel group, the percentage areas of others, which are not the focus of this study, were set as the baseline group to avoid perfect collinearity. The regression result indicates that the ignition probability will be significantly higher if the subcompartment has a higher proportion of pine or fir. Broadleaf species are believed to be fire-resistant in this region. Our result indicates that broadleaf species do contribute to a lower ignition probability than the baseline group, but the effect was not statistically significant. Stands with older trees are also less likely to have fire occurrence. Of the topographical variables, elevation was not included due to multicollinearity with proportion of fir and broadleaf, slope and distance to nearest road. Slope and aspect were both significant. For the weather group, the negative correlation of daily average temperature with fire occurrence reflects an inter-seasonal difference. In this region, fires are more likely in winter and spring due to dry weather conditions. Relative humidity has a substantially negative correlation with fire occurrence, as expected. For human activity, longer distances to the nearest main settlements indicated lower accessibility and consequently lower probability of human-induced fire. Population density and distance to nearest road were not significant.

### Factor contribution to fire size

The final model averaged from 10 RF runs explained 55.60% of the total variance, with a mean square error of 10622.55. The correlation coefficient between the observed fire sizes and the model-predicted sizes is 0.808. The 12 variables selected to build the final RF model are shown in descending order according to their % IncMSE in Table 3. Among the top five variables, there are two fuel variables (proportion of pine and broadleaf), two human activity variables (population density and distance to nearest road), and one topographical variable (slope). The overall results indicate that fuel variables contribute the most in explaining fire size, human activity comes second, while climatic variables contribute the least.

**Table 3:** Variables of RF model.

Variables	Avg % IncMSE
Proportion of pine (%)	12.3456
Population density (per/km^2^)	11.2011
Slope (**degrees**)	7.0304
Proportion of broadleaf (%)	6.5546
Distance to nearest road (km)	3.2076
Aspect (**degrees**)	2.0139
Dominant species age (yr)	1.2682
Proportion of fir (%)	1.0843
Distance to nearest settlement (km)	0.8578
Wind velocity (m/s)	0.7214
Daily average relative humidity (%)	0.3434
Daily maximum temperature (°C)	0.1300

In descending order of importance based on % IncMSE (mean decrease in accuracy) from 10 RF models. Additional estimation results using generalized adding models with incorporating interaction items are also provided in S1 Text.

### Burn distribution and factor contribution

**Fig 3:**
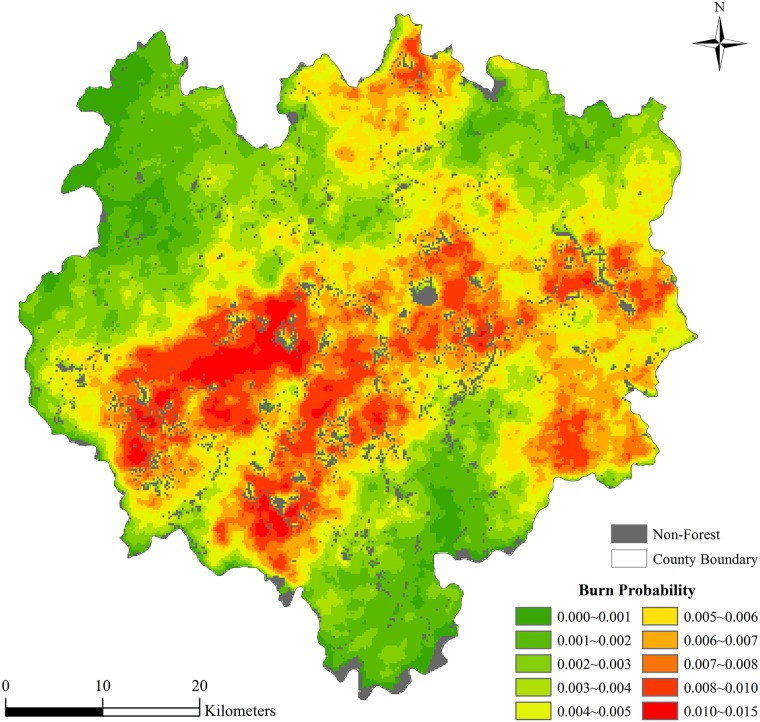
The burn probability for the study area estimated by simulation.

The BP result for each individual pixel was derived by averaging the total number of times that it burned in the 10,000 runs of simulation, and ranged from 0 to 0.014 (Fig 3), with mean and median values of 0.0047 and 0.0046, respectively. Our simulations were found to accurately predict the burning probability in the study area, as no statistical differences were found between the historical and simulated fire events (Kolmogorov-Smirnov Two-Sample Tests, p-value = 0.180). In particular, the annual average area burned by historical and simulated fire were very similar (5.41 hectares per year and 5.07 hectares per year).

**Fig 4:**
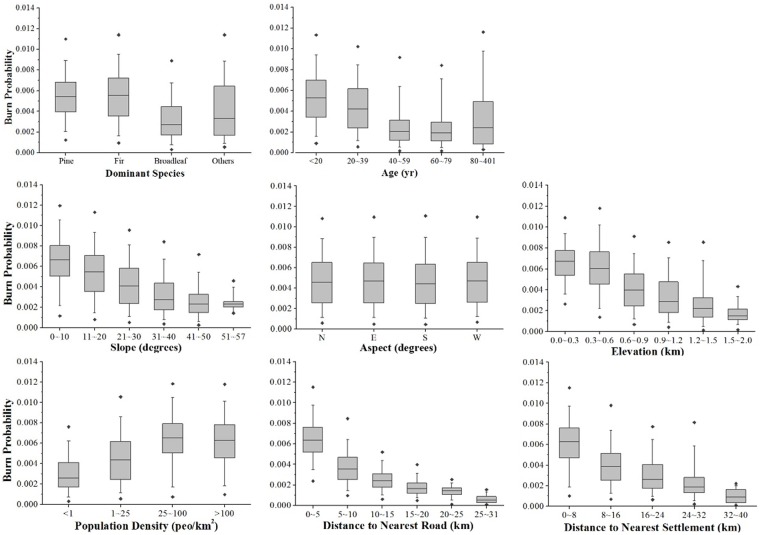
Box plots of different factors influencing burn probability. Error bars show 5th% and 95th% percentile of pixels in each bin. Cross symbols show 1st% and 99th% percentile. Fuel, upper row; Topography, center row; Human activity, lower row. Statistics were derived from a full sample of 43526 pixels in the region.

Spatially, the distribution of the BP is highly consistent with those of the elevation, roads and settlements, indicating a strong influence of topography and human activity. This observation is further confirmed by the statistical relationships of the BP with the different factors (Fig 4). In the box plots, the error bars show the 5th and 95th percentile of the pixels in each bin. The BP shows strong correlations with most of the factors. For fuel type, The fuel composition variables were used to assign the categories pine, fir, broadleaf or others, according to which fuel type had the largest % area in a pixel. Pine and fir are more likely to suffer from fire, while broadleaf forests are the least likely, indicating the strong preferential likelihood of fire among particular fuels. Forest stands at ages between 40–80 years show the least likelihood of being burnt. Topography exerts a significant influence on the BP. Both elevation and slope show negative correlations with the BP, but there is little difference by aspect. Human activity also has a strong influence on the likelihood of being burned. BPs are higher in pixels where there is more intensive human activity, e.g., higher population density, smaller distance to nearest road or smaller distance to nearest settlements.

The result of factor contribution analysis to BP was listed in Table 4. The independent variable was the logarithm of the BP due to its non-negative values. Most of the fuel, topography and human activity variables were included in the model, and all were statistically significant. Among the variables considered, distance to nearest road contributes the most in explaining the variance (42.9%). Distance to nearest settlement was not used as it is highly correlated with distance to nearest road. Elevation comes in second place, with 13.1%. Contributions from other factors were all statistically significant but with modest values <10%. The relative contribution of the factors can be derived by summing the relative importance of related variables. Therefore, the relative contributions of human activity, topography and fuel are 42.9%, 18.6% and 14.8%, respectively. Fire weather variables, which were subject to daily and seasonal variation, were not explicitly introduced into the model, as the BP is the annual average likelihood of being burned.

**Table 4:** Factors’ relative contribution to BP.

Variables	B	S.E.	Sig.	Relative importance^a^
Constant	-4.59	4.20E-03	0.000	
**Fuel**				**0.148**
Proportion of pine (%)	1.48E-02	6.23E-03	0.018	0.007
Proportion of fir (%)	2.50E-01	4.10E-03	0.000	0.029
Proportion of broadleaf (%)	-8.48E-02	4.75E-03	0.000	0.057
Dominant species age (yr)	-5.32E-03	1.06E-04	0.000	0.054
**Topography**				**0.186**
Slope (degrees)	-5.39E-03	1.51E-04	0.000	0.054
Elevation (m)	-4.04E-04	4.58E-06	0.000	0.131
**Human activity**				**0.429**
Distance to nearest road (km)	-7.13E-02	2.43E-04	0.000	0.429
			**R**^**2**^	**0.762**

^a^Relative importance is the decomposed R^2^ using *lmg* metrics with the *relaimpo* package in R v3.2.4.

## Discussion

Our results suggest that, in this region, the relative contribution of the studied factors (including fuel, topography and human activity) differs considerably among fire ignition, fire size, and BP. This finding confirms the need to analyze the holistic influence of these factors on fire likelihood. For ignition, our results based on the estimated odds ratio show that fuel type exerts the largest influence on ignition probability, followed by fire weather and human activity, while topography contributes the least, agreeing with the findings of related studies in the Mediterranean [13] and Alps regions [12]. Our result also agrees to Liu et al.’s findings in Northeast China that aspect, vegetation type and distances to roads and settlements are found to be the spatial controls of occurrence of human-caused fires [6].

For fire size, the fuel composition again topped the relative importance, providing strong evidence of preferential burning, while the influences of topography and fire weather were modest. The result agrees to the findings of the existing literature. For instance, vegetation and topography are found to top the relative importance to fire size in western United States [7]. The descending contribution to fire size of fuel, topography and weather is also found in Northeast China [19] when fire sizes are smaller than 100 ha. Study on fire size in interior Alaska also showed that human-caused fires are generally small in size and occur frequently with flammable fuel types [58]. As Liu et al. has indicated in their discussion [19], the influence of fire weather is more likely to be revealed with large-sized fires. In the context of our limited spatial scope, differences in fire weather reflect the seasonal variation in ignitions rather than their spatial variation.

The most important determinant of the fire likelihood (BP) in this subtropical coniferous forest was human activity. This finding reflects the local features of a rapid forest production cycle and strong human disturbance. By contrast, fuel composition, the factor with the largest contribution to explaining both fire occurrence and fire size, shows only a modest influence on the distribution of the BP, ranking third in terms of relative contribution. There are two possible reasons for this. On the one hand, ignitions and fire size are two variables of a single fire event, whereas burn probability represents the consequence of bulk fire events [21]. The influence of human activity was amplified through the process of overlapping repeated ignition and spreading events. On the other hand, human activity in terms of population density and distance to nearest road/settlement exhibits a strong spatial gradient. By contrast, fuel distribution is quite fragmented and linked to local differences in our study area. Therefore, human activity shows more power in explaining the spatial difference in the BP at the regional scale.

We also tried to extend our analysis to detect the change in relative contribution on spatial scales using moving window resampling analysis, as in [8,19]. Unfortunately, the attempt was not as successful as in the existing studies. The total variance explained fell sharply at pixel sizes beyond 500 m or 1000 m. This may again be attributed to the fragmented distribution of fuel. Although the moving average can provide a single value over a large pixel size, i.e., the mean, the underlying heterogeneity within the large pixel is substantial. Consequently, more random errors could have been introduced into the regression, reducing the total variance explained.

There is always an issue of covariance among factors and variables. In this study, there was also significant correlation among the elevation, fuel distribution, slope, and human activity distribution. This suggests that elevation is one of the critical underlying factors that drive the local distribution of fuel and human activity. Fuel distribution may depend on topography as certain tree species, particularly coniferous species, live only in certain elevation ranges. For instance, *P*. *massoniana* mainly occupies regions with elevations lower than 700 m in the lower reaches of the Yangtze River, while *P*. *taiwanensis* is found in elevation ranges of 600~1800m. Human activity distribution is also linked to topography as settlements and roads follow the contours of the valley. These facts suggest that elevation is one of the critical decisive factors that drive the local distribution of fuel and human activity. The results of our multiple linear regression only offer the ceteris paribus effect, i.e., the marginal contribution of elevation to BP when fuel composition and human activity variables are kept constant, and vice versa. Such a calculation obviously cannot accommodate the decisive influence of elevation on fuel composition and human activity. If the impact of elevation on human activity is taken into account, this could reveal a pervasive effect of topography, as in many other cases of forest fires in mountainous landscapes [59,60]. Analyzing the hierarchical structure of the environment-driven human–forest interaction in terms of fire needs improved analytical methods.

Our analysis is limited by the available data and analytical methods. The forest and fire data provided by local forest management authorities were precise and detailed. Nevertheless, we did not manage to identify fire scars for each fire event due to resolution and cloudiness issues but only determine the fuel type where each fire began based on the initial location of each fire [58]. The fuel composition of the forest that was actually burnt was not precisely known, introducing uncertainty in the fire size analysis. If precise fire scars could be included, the accuracy of the fire size analysis could be improved. Also, there is a matching issue of the historical fire records (1990–2010) and vegetation map (only for 2008). Fortunately, the replantation of burnt forest stands shall strictly follow local forestry planning maps according to our investigation. So the post-fire-replanted new trees had similar species and stand structure as that before the fires. In addition, 93% of fires had smaller size than the forest stand ignited, and not all the stands ignited were post-fire replanted. Dominant species age in fuel data also indicated that 166 out of 255 ignited forest stands were planted before the year of burning. As a result, the use of static vegetation map of year 2008 have only limited impact on our results.

In our analytical framework, the BP represents the annual average likelihood of being burned. Therefore, climatic variables on an annual basis should be considered, instead of fire weather variables, which are subject to monthly variation. Unfortunately, in our limited study area, the climatic conditions did not show substantial spatial differences and consequently were not introduced into the relative contribution analysis. This issue can be solved by applying the framework in a study area large enough to accommodate heterogeneous climatic conditions, or under climate change scenarios to allow the explicit inclusion of climatic variables into the relative contribution analysis.

## Conclusions

BP is a systematic measure of the likelihood of fire based on a thorough understanding of fire regime attributes and stochastic simulation. Detecting the factors affecting the BP, and their relative contribution, can provide critical information for fire risk management. In this article, we examined the contributions of fuel type, topography, and human activity to fire occurrence, size distribution and BP. The results indicate that all three factors have a significant influence on all three outcomes, but their relative contributions are substantially different for each outcome. Forest-stand fuel composition is the factor with the largest contribution to explaining fire occurrence and fire size, but the human activity factor makes the largest relative contribution to explaining the BP. Our results indicate that the non-linear relationship among fire ignition, spreading and the final BP could amplify or attenuate the contribution of individual factors. Therefore, understanding the factor contribution to the BP should provide critical information for fire risk management. Similar examinations in other study areas with different climax vegetation and human disturbance intensity will shed further light on our understanding of the drivers of fire likelihood, particularly for the issues of up-scaling and the incorporation of climatic factors.

## Supporting information

S1 Dataset Minimal sample data set of the study. (Arcmap.shp file)
(RAR)

S1 Fig Percentage of ignitions recorded by cause. *“Grave visits” means the Chinese tradition of burning paper or incense at grave sites.
(TIF)

S2 Fig Historical monthly ignition frequency. (TIF)

S3 Fig Historical ignitions. (TIF)

S4 Fig The number of simulated ignitions. Simulated ignitions are shown as number of ignitions in 10,000 years.
(TIF)

S5 Fig Cumulative distribution functions of historical fire size and simulated fire size. (TIF)

S1 Text Additional estimation results using generalized adding models in fitting fire frequency and size. (PDF)
