# Supplementary figures and images for: Factor contribution to fire occurrence, size, and burn probability in a subtropical coniferous forest in East China
Source: PLoS One. 2017 Feb 16;12(2):e0172110. doi: 10.1371/journal.pone.0172110 (PMC5313183; doi:10.1371/journal.pone.0172110)

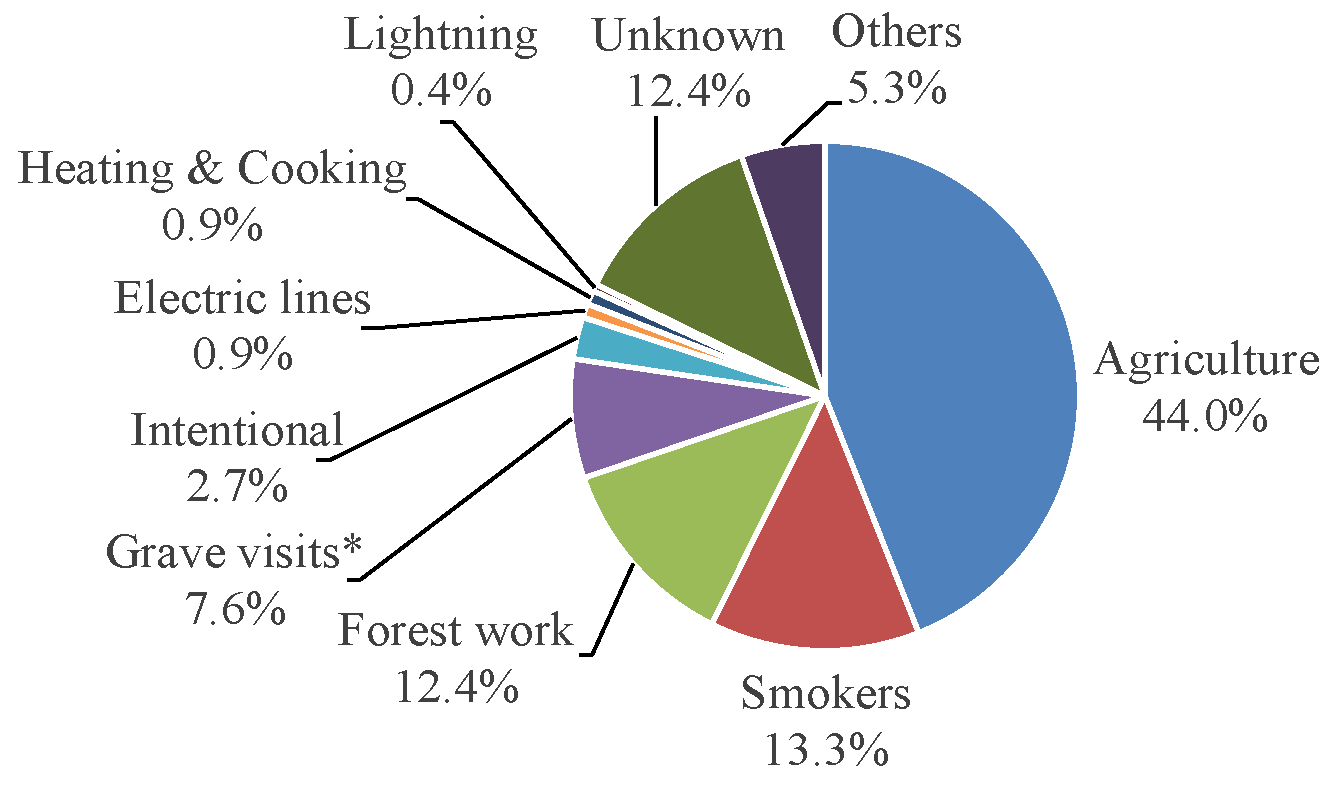

Supplement: S1 Fig — *“Grave visits” means the Chinese tradition of burning paper or incense at grave sites. (TIF) [file pone.0172110.s002.tif]

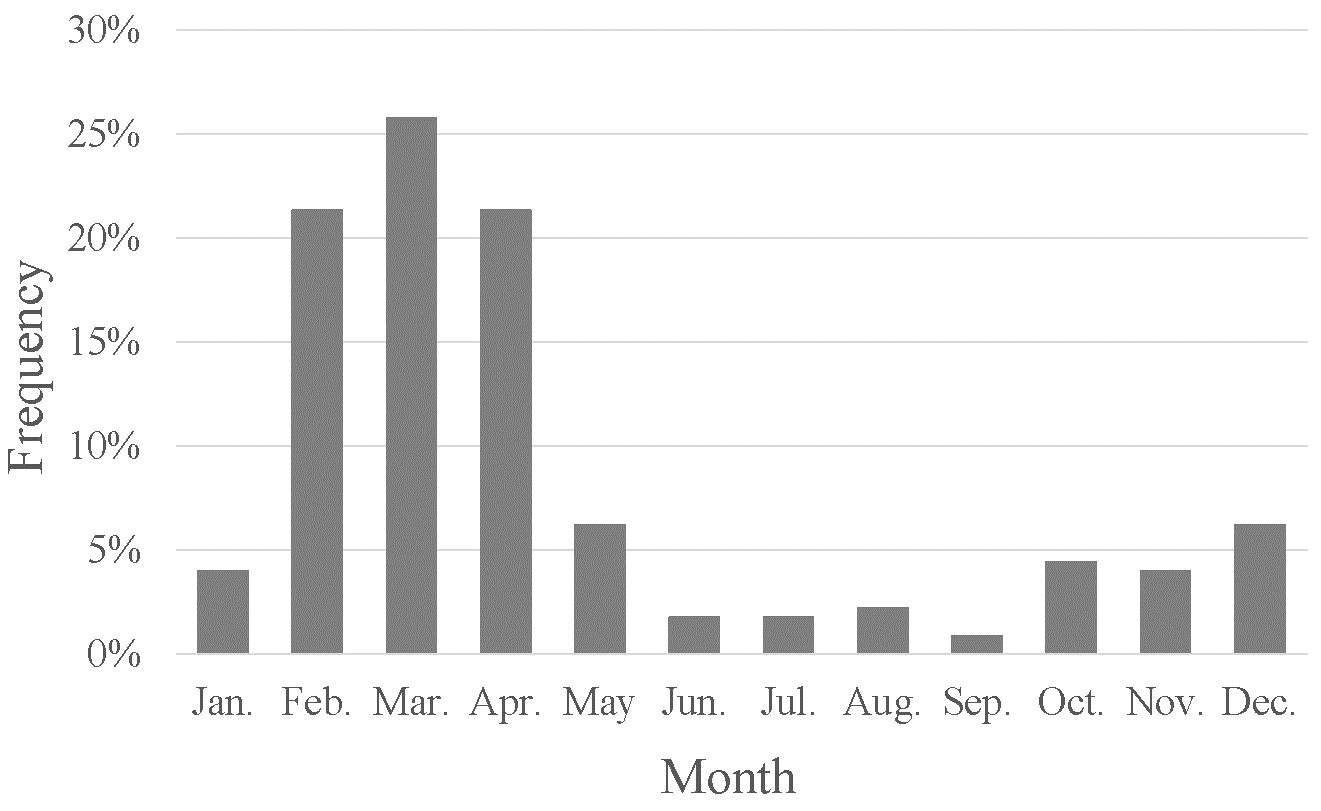

Supplement: S2 Fig — (TIF) [file pone.0172110.s003.tif]

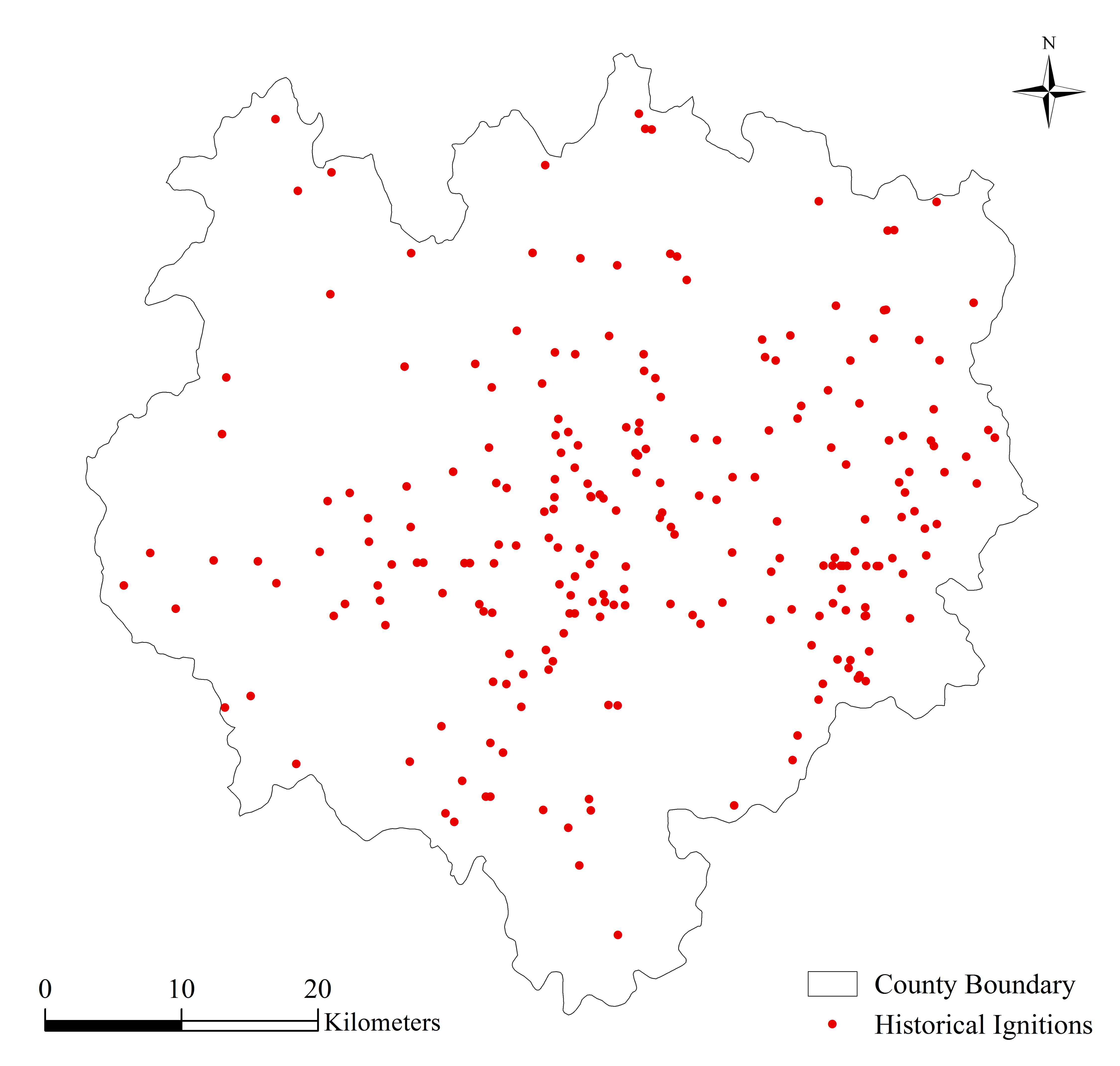

Supplement: S3 Fig — (TIF) [file pone.0172110.s004.tif]

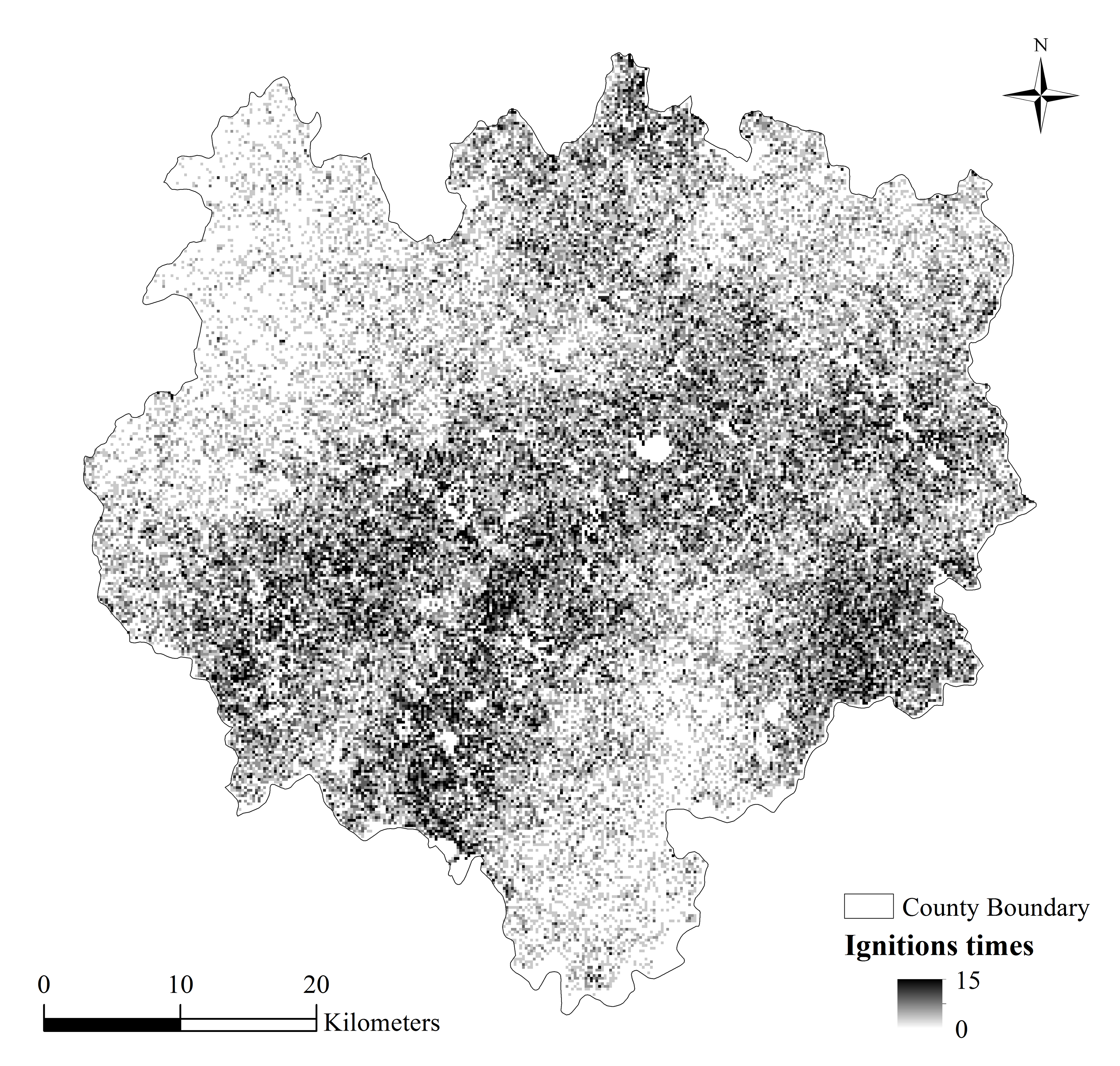

Supplement: S4 Fig — Simulated ignitions are shown as number of ignitions in 10,000 years. (TIF) [file pone.0172110.s005.tif]

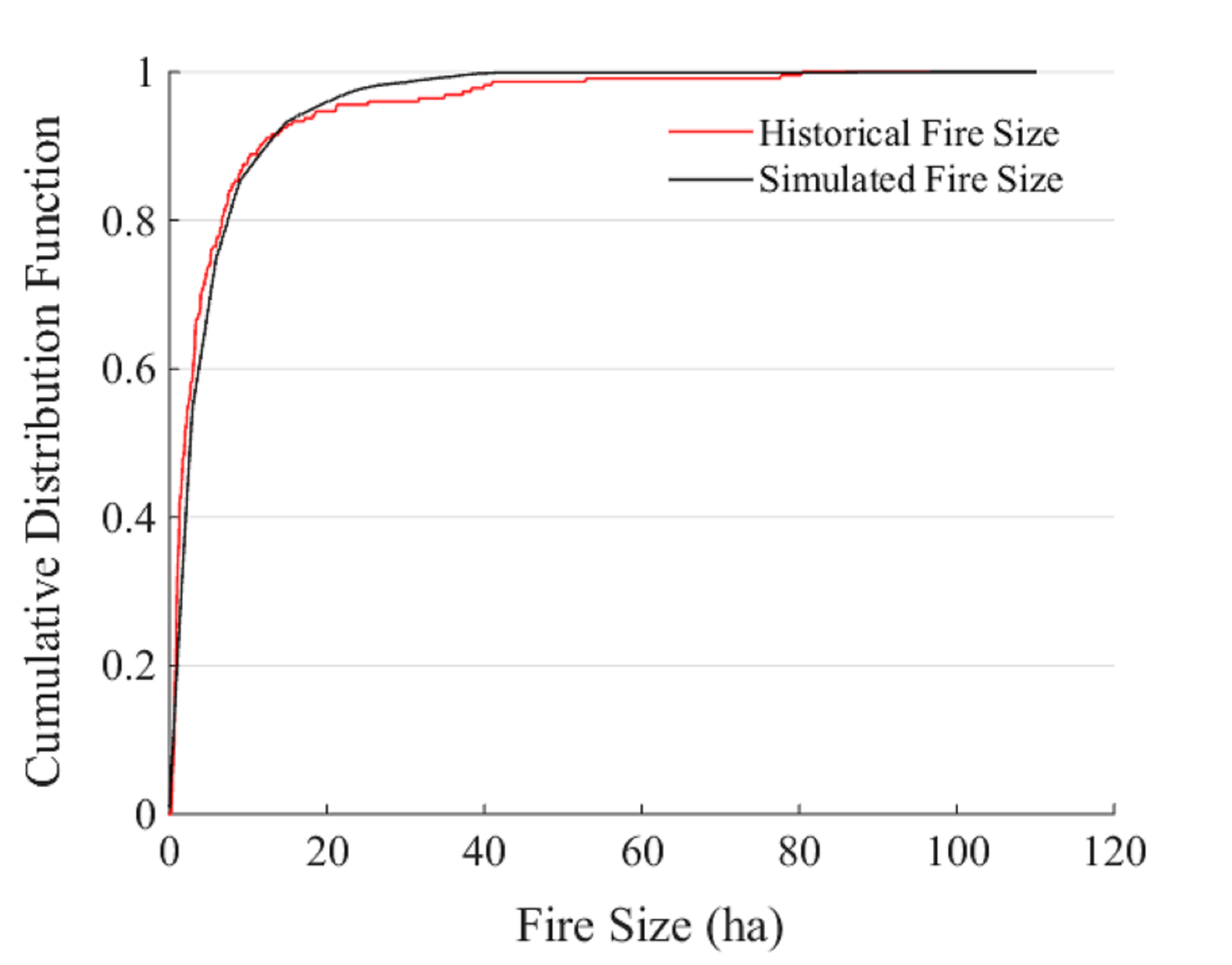

Supplement: S5 Fig — (TIF) [file pone.0172110.s006.tif]
