# Supplementary material for: Factor contribution to fire occurrence, size, and burn probability in a subtropical coniferous forest in East China
Source: PLoS One. 2017 Feb 16;12(2):e0172110. doi: 10.1371/journal.pone.0172110 (PMC5313183; doi:10.1371/journal.pone.0172110)
Supplement: S1 Text — (PDF) [file pone.0172110.s007.pdf]

Supplementary Material

When estimating factor contribution to to fire occurrence and fire size, the generalized additive models (GAMs) was also considered as suggested by one of the reviewers. GAMs provide much flexibility and are often useful when relationships between the variables are non-linear or asymmetric and not easily fitted by parametric models. A data dredging analysis was applied to run the GAMs for valid combinations of environmental predictors to identify the interactions between factors and to assess how well each model was supported by the data with the multi-model inference method for fire occurrence and fire size analysis. Function ‘dredge’ was available in MuMIn package in R v3.2.4.

In the additional analysis, several experiments based on GAMs model were conducted as suggested by the reviewer.

(1) Using GAMs to fit fire occurrence and fire size with variables shown in the current manuscript (Table 2 and Table 3).

(2) Using GAMs to fit fire occurrence and fire size with interactions slope*elevation, or fuel type*human activities.

(3) Using “dredge” method to explore the best fitted GAMs model for fire occurrence and fire size.

R^2 was used as a simple measure for the comparison among model settings. Below is a summary of all experiments:

Table S1. The results of GAMs model experiments.

**Model setting**	**Fire occurrence<br>Family= binomial<br>Link = logit func.**	**Fire size<br>Family= Gaussian<br>Link = identity**
**GAM present setting**	R^2(adj.) = 0.457	R^2(adj.) = 0.175
**GAM interaction (fuel*human activity)**	R^2(adj.) = 0.47<br>Interaction not sig.	R^2(adj.) = 0.315
**GAM interaction (slope*elevation)**	R^2(adj.) = 0.456<br>Interaction sig. (0.1 level)	R^2(adj.) = 0.17
**GAM interaction (both interaction)**	R^2(adj.) = 0.467<br>Fuel*human sig. (0.1 level)<br>Slope*elev. sig. (0.05 level)	R^2(adj.) = 0.324<br>Fuel*human sig. (0.01 level)<br>Slope*elev. No sig.
**Dredge GAM (no interaction)**	R^2(adj.) = 0.45	R^2(adj.) = 0.184
**Dredge GAM (both interactions)**	R^2(adj.) = 0.456<br>Fuel*human sig. (0.1 level)<br>Slope*elev. sig. (0.05 level)	R^2(adj.) = 0.293<br>Fuel*human sig. (0.01 level)

When compare the results of the modified models and estimation method, we found that for fire occurrence, only marginal increases in variance explained can be obtained (the R^2 of the binary logistic model presented in the manuscript is 0.45). For fire size, introducing interaction fuel*human activity can substantially increase the R^2, but the total variance explained is still small compared to the result derived by RF. Given the tests above, we have decided to keep the result of binary logistic regression and random forest in analyzing fire occurrence and fire size, respectively. But the results listed above can be of value for model fitting in other regions and future research.
